# Severe choline deficiency induces alternative splicing aberrance in optimized duck primary hepatocyte cultures

**DOI:** 10.5713/ab.22.0051

**Published:** 2022-05-02

**Authors:** Lulu Zhao, Hongying Cai, Yongbao Wu, Changfu Tian, Zhiguo Wen, Peilong Yang

**Affiliations:** 1 Institute of Feed Research, Chinese Academy of Agricultural Sciences, Beijing, 100081, China; 2 State Key Laboratory of Agrobiotechnology, College of Biological Sciences, China Agricultural University, Beijing, 100083, China

**Keywords:** Alternative Splicing, Choline-deficient Model, Lipid Metabolism, Primary Duck Hepatocyte

## Abstract

**Objective:**

Choline deficiency, one main trigger for nonalcoholic fatty liver disease (NAFLD), is closely related to lipid metabolism disorder. Previous study in a choline-deficient model has largely focused on gene expression rather than gene structure, especially sparse are studies regarding to alternative splicing (AS). In modern life science research, primary hepatocytes culture technology facilitates such studies, which can accurately imitate liver activity *in vitro* and show unique superiority. Whereas limitations to traditional hepatocytes culture technology exist in terms of efficiency and operability. This study pursued an optimization culture method for duck primary hepatocytes to explore AS in choline-deficient model.

**Methods:**

We performed an optimization culture method for duck primary hepatocytes with multi-step digestion procedure from Pekin duck embryos. Subsequently a NAFLD model was constructed with choline-free medium. RNA-seq and further analysis by rMATS were performed to identify AS events alterations in choline-deficency duck primary hepatocytes.

**Results:**

The results showed E13 (embryonic day 13) to E15 is suitable to obtain hepatocytes, and the viability reached over 95% by trypan blue exclusion assay. Primary hepatocyte retained their biological function as well identified by Periodic Acid-Schiff staining method and Glucose-6-phosphate dehydrogenase activity assay, respectively. Meanwhile, genes of *alb* and *afp* and specific protein of albumin were detected to verify cultured hepatocytes. Immunofluorescence was used to evaluate purity of hepatocytes, presenting up to 90%. On this base, choline-deficient model was constructed and displayed significantly increase of intracellular triglyceride and cholesterol as reported previously. Intriguingly, our data suggested that AS events in choline-deficient model were implicated in pivotal biological processes as an aberrant transcriptional regulator, of which 16 genes were involved in lipid metabolism and highly enriched in glycerophospholipid metabolism.

**Conclusion:**

An effective and rapid protocol for obtaining duck primary hepatocytes was established, by which our findings manifested choline deficiency could induce the accumulation of lipid and result in aberrant AS events in hepatocytes, providing a novel insight into various AS in the metabolism role of choline.

## INTRODUCTION

Nonalcoholic fatty liver disease (NAFLD), a growing public health concern worldwide, has a disease spectrum ranging from simple steatosis to nonalcoholic steatohepatitis, liver fibrosis, cirrhosis, and hepatocellular carcinoma [1,2]. NAFLD is often closely associated with obesity and metabolic disorder syndrome, increasing with growing problem of obesity in the general population. Till now, the pathogenesis of NAFLD is not completely understood. Nowadays, increasing number of liver functional research findings both *in vitro* and *in vivo* are established in mammals, especially focusing on human, mouse, and rat, while less in poultry. Distinguished from mammalian species where fat synthesis mainly occurs in liver and adipose tissue, fat of poultry is mainly synthesized de novo by the liver, which is a central/vital organ function in lipid metabolism [3,4]. What’s more, compared with other terrestrial poultry (chicken, turkey, and others), waterfowl are equipped with high liver lipid storage capacity that is shown by their response to force feeding. Lu et al [5] previously reported that deletion of *leptin* gene is one reason for the powerful liver in geese with their prediction by application of comparative genomics, but they are still not fully aware of what is the trigger and molecular mechanisms underlying the finding. Pekin duck as a waterfowl is regarded a fascinating study object in liver fat metabolism research.

Hepatocytes culture models *in vitro* rather than an *in vivo* organism models have been used extensively for animal virological and biomedical research [6]. Most importantly, on account of primary hepatocytes cultured technology can accurately imitate liver activity *in vivo*, it is superior to *in vivo* models. The primary culture of human hepatocytes is an important tool in the field of toxicity studies, evaluating drug-metabolizing ability, and design of bioartificial liver devices [7–9]. The current classic method called two-step collagenase perfusion technique introduced by Seglen in the 1970s is frequently applied in isolating primary hepatocytes from adult individuals [10]. Meanwhile, enzymatic digestion is also an available alternative to obtain cell suspension using collagenase or trypsin. Nevertheless, because of the high cost, strict operational technical demands and other aspects, there are some limitations in the practical application of the traditional perfusion technique. In addition, the process of digesting cells may also affect their viability in enzymatic methods. Some researchers adopted individual optimization method based on previous approach to obtain primary hepatocytes [11,12]. In the same vein, the protocol for obtaining duck primary hepatocytes is technically challenging.

Establishment of NAFLD models are roughly divided into two categories: gene knockout or individuals with a mutation affecting oxidation of fatty acids in the liver, and unbalanced fatty acid synthesis and oxidation in the liver caused by diet and drugs [13–16]. Cohort studies indicated that fatty liver development is nutritionally induced by choline deficiency [17,18]. Choline is an essential nutrient for animal and human and also occurs in a list of ingredients in basal medium [19,20], which is involved in some crucial processes such as the biosynthesis of neurotransmitter acetylcholine and the major membrane component phosphatidylcholine (PC). Previous analysis of gene expression in choline-deficient models has largely focused on gene expression rather than gene structure, especially sparse are studies regarding alternative splicing (AS). AS is a post-transcriptional level during mRNA processing and plays an important role in gene regulation relying on cis-acting and trans-acting elements, which gives rise to varying combinations of exon inclusion and splice site usage that produces multiple transcript isoforms [21,22]. Aberrant splicing underlies many pathological processes, as a large percentage of disease mutations disrupt splicing and generate aberrant gene products. An alternative splicing variant of APPL1 (APPL1sv) that is highly expressed in mouse liver, pancreas, and spleen tissues suppresses hepatic adiponectin signaling and function in a mouse model of obesity and diabetic dyslipidemia [23,24]. In cancer research, high AS events are more prevalent in tumor relative to matched normal tissues [25].

Herein we describe a method for the isolation and culture of duck primary hepatocytes by performing an optimized enzymatic digestion technique, which involves comparing with different embryonic ages of Pekin duck and digestion approaches and is a rapid and efficient method for obtaining duck primary hepatocytes with high purity and biological activity checked both morphologically and functionally. Based on the method of isolation and culture of primary hepatocytes, we explored lipid metabolic alterations in hepatocytes with choline-free medium and constructed NAFLD model. Moreover, the study based on RNA-seq analysis aimed to explain AS variation in choline-deficient model and its potential contribution towards understanding lipid metabolism.

## MATERIALS AND METHODS

All animal experimental procedures were approved by the Animal Ethic Committee of the Chinese Academy of Agricultural Sciences (CAAS) and performed according to the guidelines for animal experiments set by the National Institute of Animal Health (Statement No. AEC-CAAS-20200506).

### Hepatocyte isolation and culture

Hepatocytes were isolated from different ages of Pekin duck, ranging from E11 (embryonic day 11) to E20. Firstly, embryonated duck eggs were cleaned with iodophor and 75% ethanol. Then livers were removed from duck embryos in sterile conditions followed by gallbladder evisceration. Simultaneously remove the adherent loose connective tissue and liver capsule around the liver. Special care must be given when resecting the liver. The small pieces of liver tissue obtained were soaked in Dulbecco’s Hanks balanced salt solution (D-Hank’s) to diminish blood cells and adherent non-hepatocytes, then repeat the above steps three times. The multi-step digestion method was performed as follows. Pieces of liver tissues were collected and digested using trypsin-trypsin with ethylenediaminetetraacetic acid (EDTA) (0.25%) (Gibco, Grand Island, NY, USA) for 15 to 30 s. After the tissues were softened and whitened, wash them three times with Dulbecco’s modified eagle medium (DMEM) (Gibco, USA). Subsequently, tissues were transferred to a sterile centrifuge tube, and this was followed by cutting then into 1 mm^3^ pieces with a scalpel, washing twice with DMEM. The supernatant was removed after the process of cells self-sedimentation. Remaining tissue block was separated fully using pipette with trypsin-EDTA (0.25%) within 30 s until digesting into cell suspension. One out of ten volume of fetal bovine serum (Gibco, USA) was used to terminate the trypsin digestion. The mixture liquid was centrifuged at 500 g for 5 min at 4°C, which the upper was discarded and the cells were washed with DMEM. This process is repeated twice. The obtained cells were resuspended with complete medium (high glucose medium DMEM enriched with 10% fetal bovine serum, penicillin 10^5^ U/L (Gibco, USA), streptomycin (Gibco, USA) 100 mg/L, and were passed through a 70 μm cell sieve (Corning, NY, USA). Counted hepatocytes were seeded into the culture flask at a density of 1×10^6^ cells/mL. Cells were cultured at 37°C under an atmosphere of 5% CO_2_. After seeding 12 h, cells were refreshed with new medium. While cells were cultured with choline-free medium in choline-deficient model.

### Hepatocyte viability assay

The cell suspension is mixed with 0.4% trypan blue solution at a ratio of 9:1 (final concentration 0.04%), and stained for 3 min. An appropriate number of stained cells were observed by light microscopy and counted with a hemocytometer. Dead cells are blue, swollen, and dull; living cells are not colored and maintain normal shape, shiny. Cell survival rate (%) = total number of living cells/(total number of living cells+total number of dead cells)×100%.

Growth curve assay is a common method to determine the absolute number of cell growth, which is also an important indicator of cell viability and is one of the basic parameters of the biological characteristics of cultured cells. Cell viability and proliferation was measured using MTT method [26]. Hepatocytes were counted, resuspended at 2×10^5^ cells per 200 μL medium, and plated in 96-well plates for incubation at 37°C under an atmosphere of 5% CO_2_. After 12 h, the supernatant was replaced with fresh medium. At the end of the incubation, 10 μL of 5 μM MTT reagent (Sigma-Aldrich, St Louis, MO, USA) was added to each well, and incubated for 4 h. Next, the medium was removed and MTT precipitate was solubilize by the addition of 200 μL dimethyl sulfoxide per well. The absorbance at 490 nm was measured in triplicate wells. The cell growth curve was drawn based on the data, showing whether isolated cells have typical cell growth characteristics.

### Functional assay

We used the Periodic Acid-Schiff staining method to evaluate the biological function of primary hepatocytes through the cell glycogen content. After reaching confluence, primary hepatocytes were fixed in Periodic Acid Schiff (PAS) fixative solution and then stained with PAS for subsequent examination under a light microscope. At the same time, a negative control was set up where the same cells were treated identically, only without oxidizing agent. The PAS yielded signal was captured in full colour using bright field [27].

Glucose-6-phosphate dehydrogenase (G6PDH) activity reflects biosynthesis of an individual to some extent as well. Isolated hepatocytes were collected and then broken by an ultrasonic cell-crushing device in an ice bath. The supernatants were collected after centrifugation at 8,000 g, 4°C for 10 min for G6PDH assay. Finally, G6PDH were detected monitored by the production of NADPH with a consequent increasing in absorbance at 340 nm.

### Preliminary identification of isolated cells at mRNA level

Albumin (ALB) is secreted by liver cells. Alpha fetoprotein (AFP) is normally derived from embryonic liver cells. The isolated cells were preliminarily identified by *alb* and *afp* genes at mRNA level. Total RNA was extracted using the *TRIzol* RNA extraction reagent (Invitrogen, Grand Island, NY, US) from hepatocytes. cDNA was synthesized using a cDNA kit (TransGen, Beijing, China). The *alb* and *afp* genes were amplified by polymerase chain reaction (PCR), and then the amplified gene sequence was sequenced to be aligned in NCBI (https://www.ncbi.nlm.nih.gov). *Alb* is located on the fourth chromosome, the gene accession number: NM_00 1310394.1, from 46869333 to 46879914. *Afp* is also located on the fourth chromosome, with gene accession number: NC_040049.1, from 46836428 to 46864918. Primers were designed as follows. ALB-F: ATGAAGTGGGTAACATTA ATTTC, ALB-R: TTAAGCACCAATTCCTAATGT. Primers: AFP-F: ACTGTAGTCAAAGCCCTGC, AFP-R: TTGGAA TCAATCCTCTTTCACAAA.

### Immunofluorescence assay

Isolated hepatocytes were fixed with 4% paraformaldehyde for 30 min and permeabilized with 0.2% Triton X-100 in phosphate-buffered saline (PBS) for 10 min. Then cells were incubated with the polyclonal antibody to ALB serum (Origene, Rockville, MD, USA; AP21444SU-N, 1:100 dilutions) for 1 hour at 37°C and washed three times with PBS. The cells were incubated afterward with donkey anti-goat immunoglobulin G HandL conjugated to fluorescein isothiocyanate (FITC) (Abcam, Cambridge, MA, USA; ab6881, 1:200 dilution) for 1 hour at 37°C and washed three times with PBS. Subsequently, cells were incubated with 4′,6-diamidino-2-phenylin-dole (DAPI) for staining of nuclei (Soledad Bao, Beijing, China). Finally, the anti-fluorescence quencher was used to seal the sections. The stained cells were observed under the fluorescence microscope (Nikon, Tokyo, Japan).

### Detection of triglyceride and total cholesterol

Under different choline levels treatments for 24 h, samples were collected including cells pellet (intracellular) and supernatant (extracellular) fractions, separately. Cells were rinsed and resuspended with PBS and then broken by an ultrasonic cell-crushing device in an ice bath with 300 μL PBS. The content of triglyceride (TG) and total cholesterol (T-CHO) were measured in the fractions of freshly obtained samples with commercial kits according to the manufacturer’s instructions (Jiancheng, Nanjing, China). The levels of TG and T-CHO were normalized by total protein amount.

Meanwhile, to determine whether changes in the viability of hepatocytes are caused by choline deficient conditions, hepatocyte viability was measured with MTT assay under different choline levels treatments for 12 h and 24 h respectively.

### RNA-seq: library preparation, Illumina Hiseq xten/Nova seq 6000 sequencing and read mapping

RNA-seq transcriptome library was prepared following TruSeqTM RNA sample preparation Kit from Illumina (San Diego, CA, USA) using 1 μg of total RNA. Shortly, messenger RNA was isolated according to poly A selection method by oligo (dT) beads and then fragmented by fragmentation buffer. Secondly double-stranded cDNA was synthesized using a SuperScript double-stranded cDNA synthesis kit (Invitrogen, Carlsbad, CA, USA) with random hexamer primers (Illumina, USA). Then the synthesized cDNA was subjected to end-repair, phosphorylation, and ‘A’ base addition according to Illumina’s library construction protocol. Libraries were size selected for cDNA target fragments of 300 bp on 2% low range ultra agarose followed by PCR amplified using Phusion DNA polymerase (NEB, Ipswich, MA, USA) for 15 PCR cycles. After quantified by TBS380, paired-end RNA-seq sequencing library was sequenced with the Illumina HiSeqxten/NovaSeq 6000 sequencer (2×150 bp read length). The raw paired end reads were trimmed, and quality controlled by SeqPrep ( https://github.com/jstjohn/SeqPrep) and Sickle ( https://github.com/najoshi/sickle) with default parameters. Then clean reads were separately aligned to reference genome with orientation mode using HISAT2 ( http://ccb.jhu.edu/software/hisat2/index.shtml) [28] software. The mapped reads of each sample were assembled by StringTie ( https://ccb.jhu.edu/software/stringtie/index.shtml) in a reference-based approach [29].

### Identification and analysis of alternative splice events

All the alternative splice events that occurred in our sample were identified by using recently releases program rMATS (http//rnaseq-mats.sourceforge.net/index.html) [30]. Only the isoforms that were similar to the reference or comprised novel splice junctions were considered, and the splicing differences were detected as exon inclusion, exclusion, alternative 5′, 3′, and intron retention events. The value of IncLevelDiff (ΔPSI) represents the extent of differences in analysis procedure. PSI (Percent spliced in) = splice_in/(splice_in+splice_out) and ΔPSI (group1/group2) = PSI_group1 - PSI_group2. Kyoto encyclopedia of genes and genomes (KEGG) analysis were performed using the free online platform of Majorbio Cloud Platform (www.majorbio.com).

### Statistical analysis

Statistical analysis of the data was performed by using the statistical software SPSS21 with the significance level set at p<0.05. The significance levels of the content of TG and T-CHO are determined by independent sample T-test. Data are presented as the mean±the standard deviation. In analysis of AS, JunctionCountOnly (JC) mode was used for the quantification. Meanwhile, probability values were adjusted for false discovery rate (FDR) and FDR<0.05 with Wilcoxon rank-sum test was considered statistically significant.

## RESULTS

### Optimization of culture protocols to isolate hepatocyte from embryonated duck

**Figure 1:**
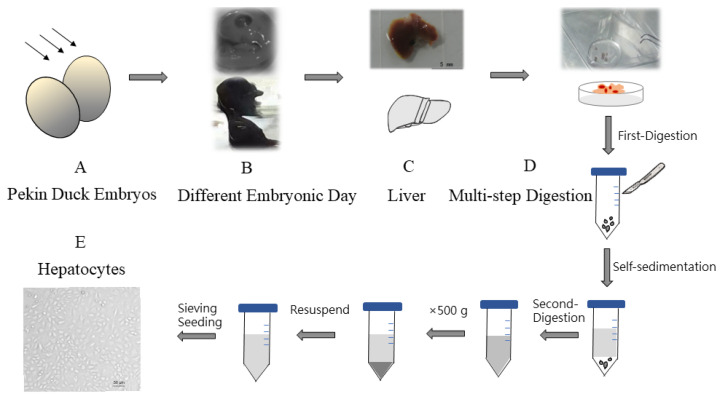
The protocols to isolate hepatocytes from embryonated duck. (A) Duck embryo viability was determined using egg candler. (B) Individuals were compared with different embryonic days. (C) Livers were obtained from B. (D) Multi-step digestion was adopted. (E) Hepatocytes were seeded and cultured.

Hepatocytes were isolated from different ages of Pekin duck, ranging from E11 to E20. And it showed differences due to ages. As the embryo grew, more and more adherent loose connective tissue occurred in liver, and the difficulty score increased. Considering operability and normal differentiation, it was not suitable to choose E11 or earlier embryonic days to obtain hepatocytes. The status of small pieces of liver tissue was monitored and multi-step digestion was adopted to avoid over-digestion, which spanned approximately 10 min throughout the digestion process (Figure 1). Cells were cultivated for 24 h, 48 h, 72 h, and 96 h, and then cell morphology was observed under a light microscopy (Supplementary Figure S1). We found that E13 to E15 is suitable to obtain hepatocytes and shortcomings are more prominent with aging.

### Identification of isolate hepatocytes viability

**Figure 2:**
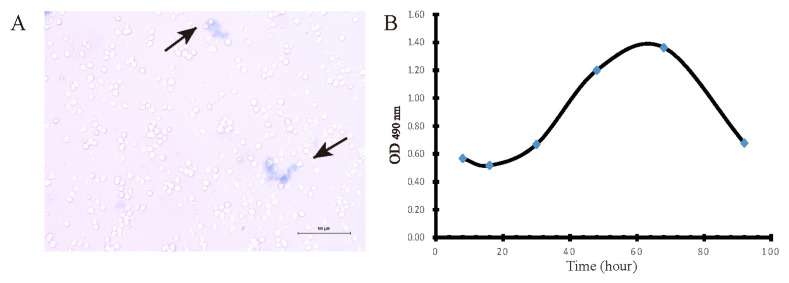
Measurement of isolate hepatocytes viability. (A) Image of Hepatocytes treated with trypan blue was captured by light microscopy (bar = 100 μm). (B) Cell growth curve was drawn by MTT assay at 490 nm absorbance, n = 6.

Hepatocyte viability was assessed using trypan blue exclusion assay under light microscopy. Dead cells are blue, swollen, and dull (black arrows); living cells are not colored and maintain normal shape, shiny. Cell survival rate reached over 95% of total number of cells, which showed that high viability hepatocytes were obtained by our optimized method. Furthermore, a cell growth curve was drawn by MTT assay and showed with four characteristic stages: latent phase (1 to 24 h after inoculum), exponential phase (24 to 48 h), plateau phase (48 to 72 h) and decline phase (72 h thereafter) (Figure 2).

### Assessment of isolate hepatocytes biological function

**Figure 3:**
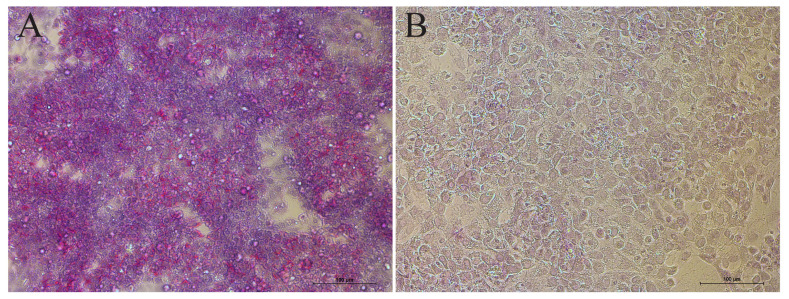
Glycogen storage of isolate hepatocytes. (A) Red and fuchsia staining indicated PASpositivity. (B) Negative control was that the same cells were treated identically without oxidizing agent only. PAS, periodic acid Schiff. Bars = 100 μm.

Hepatocyte is a cell with a high degree of differentiation. Once hepatocytes depart from liver environment, they soon lose ability of differentiation and repopulation. Subsequently some of the biological function of hepatocytes will be lost. Schiff staining was performed to evaluate the status of hepatic glycogen storage. Red and fuchsia staining indicated PASpositivity (Figure 3A). Compared with negative control (Figure 3B), it showed that the isolated hepatocyte was equipped with the favourable ability to store glycogen. Another function test was the activity of G6PDH, which displays the biosynthesis and antioxidant capacity of hepatocytes to some extent. We performed the detection together with HepG2 (a stable cell line) to reduce errors derived from the procedure. The results demonstrated that G6PDH of primary hepatocytes reached 2.9±0.13 U/10^5^ cell, approximately one third of the activity of HepG2’ (9.0±0.2 U/10^5^ cell).

### Detection *alb* and *afp* in isolate hepatocytes at mRNA level

**Figure 4:**
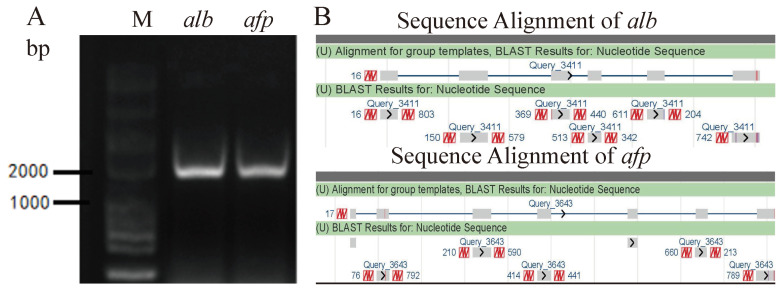
Detection alb and afp in isolate hepatocytes at mRNA level. (A) The amplified products of alb and afp were detected by agarose gel electrophoresis, respectively (B) Sequencing of alb and afp amplicons were aligned by NCBI (The upper is sequence of the genes including introns and exons in Genebank; the lower is sequencing results.).

ALB is secreted by liver cells and AFP is normally derived from embryonic liver cells. Isolated hepatocytes were preliminarily identified by PCR amplicons of these two typical genes using the cDNA as templates. The full coding sequences (CDs) length of *alb* is 1,848 bp and the full CDs length of *afp* is 1,809 bp. Nucleic acid electrophoresis indicated that of these two genes were obtained according to the base pairs of markers, which is consistent with expectation (Figure 4A). PCR product was then purified, sequenced and aligned. The results showed that *alb* and *afp* of isolated hepatocytes had been detected at mRNA level due to these sequenced regions thatspanned exon-exon junctions (Figure 4B).

### Identify the purity of isolate hepatocytes (immunofluorescent staining)

**Figure 5:**
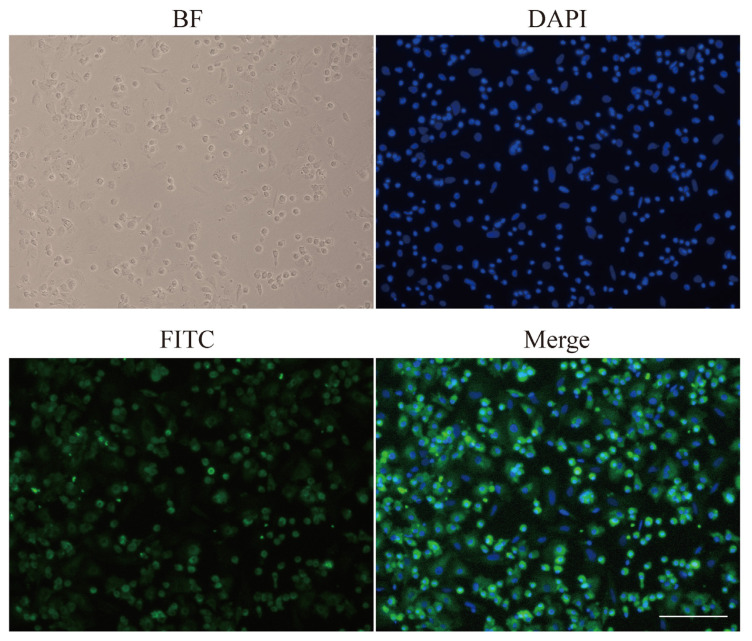
Immunofluorescent staining of ALB in isolate hepatocytes. ALB, albumin; BF, bright field; FITC, AFITC fluorescence; DAPI, 4′,6-diamidino-2-phenylindole staining; Merge, merged view of the FITC and DAPI images.

ALB is the most abundant protein in human blood and is mainly expressed by hepatocytes. In our study, ALB was detected by immunofluorescent analysis to identify the purity of hepatocytes. Brightfield image showed fixed cells. DAPI staining allowed us to focus on nuclei. FITC (green) represented the location of ALB. Furthermore, merged cells image coloured blue and green denoted hepatocytes (Figure 5). The purity of hepatocytes was up to 90% by immunofluorescent assay. Meanwhile, binuclear cells were often found, and sometimes there were multinucleated cells, which is a feature of hepatocytes.

### Determination of triglyceride and total cholesterol content in choline-deficient model

**Figure 6:**
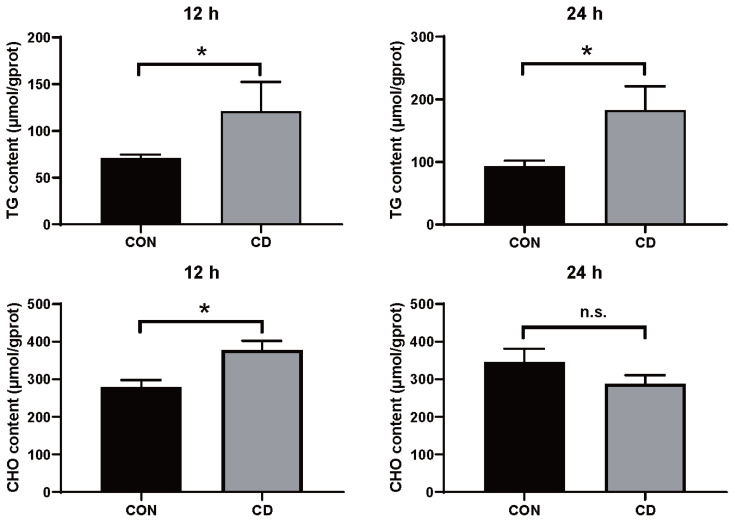
The content of TG and T-CHO altered in choline-deficient duck primary hepatocytes model. Primary hepatocytes were cultured in normal medium and choline-free medium treatment for 12 h and 24 h, respectively. Values represent the mean±SEM of two independent experiments performed in duplicate. TG, triglyceride; T-CHO, total cholesterol; SEM, standard error of the mean.* p<0.05, n = 6.

To explore the role of choline in hepatocytes culture, the content of TG and T-CHO were tested. Hepatic TG increased in choline-deficient group after treatment for 12 h significantly. Notably, the content of TG was higher in choline-deficient group after treatment for 24 h (Figure 6). Meanwhile, hepatic T-CHO was more in choline-deficient group treated for 12 h, less in choline-deficient group treated for 24 h (not significant). These results suggested that lipid metabolic alterations in choline-deficient duck primary hepatocytes model to some extent.

### Analysis of alternative splicing events in choline-deficient model

**Figure 7:**
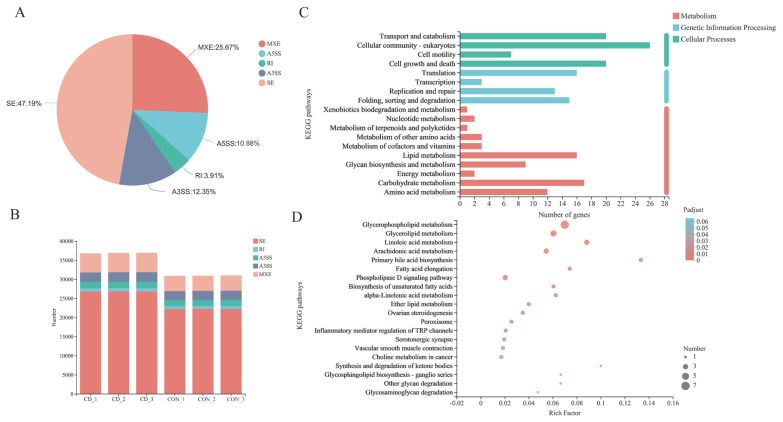
Alteration of AS events in choline-deficient group. (A) Each type of AS events differed between choline-deficient group and control group; (B) The analysis showed the AS alterations in choline-deficient model overall. (C) It revealed the AS involved genes enrichment analysis in KEGG pathway. (D) Genes-AS involved in lipid metabolism enrichment analysis in KEGG pathway (the top 20 ranked significant pathways), n = 3. AS, alternative splicing; KEGG, Kyoto encyclopedia of genes and genomes.

AS events were analyzed and divided into five basic types by bioinformatics analysis tools: Skipped exon (SE), alternative 5’splice site (A5SS), alternative 3’splice site (A3SS), mutually exclusion exon (MXE) and retained intron (RI) [31]. Overall, 36,807, 36,911, and 36,921 AS events of three parallel samples were individually identified in choline-deficient model that occurred more frequently than 30,897, 30,933, and 31,015 AS events happening in control group (Figure 7B; Supplementary Table S1). Distribution of AS events is similar in both groups: SE is the predominant type of AS and RI is least prevalent type. Each type of AS events differed between choline-deficient group and control group: the proportion of SE account for 47.19%, the proportion of MXE account for 25.67%, the proportion of A3SS account for 12.35%, the proportion of A5SS account for 10.88% and the proportion of RI account for 3.19% (Figure 7A). Subsequently, comparisons were made between 634 genes involved significant AS events that of 269 genes were up-represented and 365 genes under-represented in choline-deficient group. KEGG pathway analysis revealed that the majority of these genes were enriched in cellular processes including cellular community-eukaryotes pathway, transport and catabolism pathway and cell growth and death pathway, in metabolism including carbohydrate metabolism pathway, lipid metabolism pathway and amino acid metabolism pathway, in genetic information processing including folding, sorting and degradation pathway, replication and repair pathway and translation pathway. With lipid metabolism, the related genes were significantly enriched in glycerophospholipid metabolism (Figure 7 C and D).

**Figure 8:**
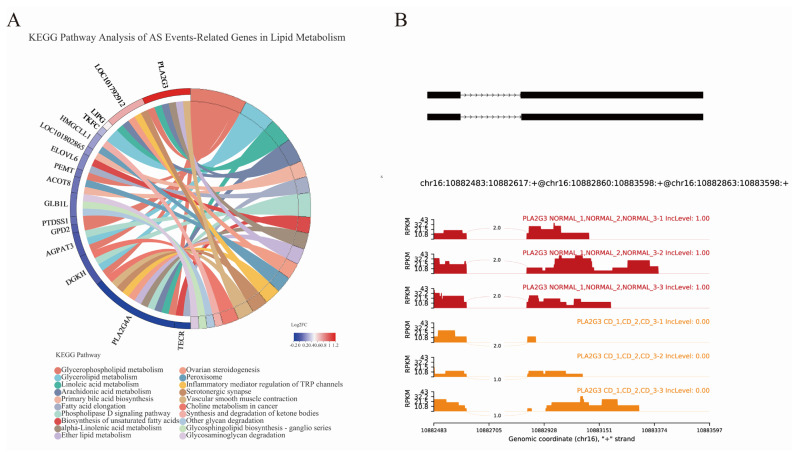
Comparation of AS-genes related lipid metabolism in choline-deficient group. (A) Significant AS events related genes in lipid metabolism were represented (log2 FC = Con/CD). (B) The AS event of PLA2G3 was drawn by rMATS, n = 3. AS, alternative splicing; FC, fold change ; CD, choline-deficient group; PLA2G3, phospholipase A2 group III, transcript variant X1.

To explore the connections of the AS events with lipid metabolism genes in choline-deficient model, we compared the related 16 genes transcriptional level and altered isoform of genes. Eight AS events of the genes belong to SE: Related genes included 3-hydroxymethyl-3-methylglutaryl-CoA lyase like 1, transcript variant X1 (*HMGCLL1*), trans-2,3-enoyl-CoA reductase, transcript variant X1 (*TECR*), diacylglycerol kinase eta (*DGKH*), 1-acylglycerol-3-phosphate O-acyltransferase 3, transcript variant X2 (*AGPAT3*), and phospholipase A2 group IVA, transcript variant X3 (*PLA2G4A*). Three AS events of the genes belong to MXE. Related genes included sterol 26-hydroxylase, mitochondrial (LOC101802865), phosphatidylserine synthase 1, transcript variant X1 (*PTDSS1*), and triokinase and FMN cyclase, transcript variant X4 (*TKFC*). Four AS events of the genes belong to A3SS. Related genes included acyl-CoA thioesterase 8 (*ACOT8*), phospholipase A2 group III, transcript variant X1 (*PLA2G3*), glycerol-3-phosphate dehydrogenase 2, transcript variant X1 (*GPD2*) and lipase G, endothelial type (*LIPG*). Four AS events of the genes belong to A5SS. Related genes included ELOVL fatty acid elongase 6 (*ELOVL6*), cytochrome P450 2J2-like, transcript variant X3 (*LOC101792912*), phosphatidylethanolamine N-methyltransferase, transcript variant X2 (*PEMT*) and galactosidase beta 1 like, transcript variant X1 (*GLB1L*) (Supplementary Table S2). Here we described the AS event of PLA2G3 and hypothesized that A3SS of PLA2G3 contributed to down-regulation of the gene due to generating transcripts harboring premature termination codons that are recognized by nonsense-mediated decay (Figure 8B).

## DISCUSSION

Primary hepatocytes are extensively used for evaluating specific liver functions. Duck hepatocyte primary cultures have been widely used for duck hepatitis B virus replication studies and antiviral screening [32]. Traditional two-step collagenase perfusion technique that is prone to suffering from failing or difficult points in portal vein cannulation is frequently applied in isolating primary hepatocytes from adult individuals with employment of pumps and other devices [33]. Results of our study indicated that our optimization method based on multi-step digestion technology could assist researchers to obtain duck primary hepatocytes without complex devices. Instead of previous enzymatic digestion method, multi-step digestion is a key process, which reduced concentration and reaction time indirectly to reduce cellular damage. Trypan blue exclusion assay showed that multi-step digestion technology was a sensible option to achieve cells of high viability. Moreover, liver not only contains parenchymal liver cells but also includes non-parenchymal cells, such as portal fibroblasts (residing in the portal niche), vascular smooth muscle cells (residing within the hepatic artery and portal vein walls), and hepatic stellate cells (located in the perisinusoidal space throughout the parenchyma) [34,35]. With the growth of embryo, the proportion of non-parenchymal cells increase in the liver. Given that the differentiation to liver lobular structures completing at E12 with further functional differentiation and increasing amount of non-parenchymal cells progressive with embryo age [36], our study pointed and confirmed that E13 to E15 is more suitable to obtain hepatocytes with high purity. In previous methods, hepatocytes were purified by centrifugal elutriation technique, differential attachment technique, multiple filtrations, and low-speed centrifugation technique [37–39]. We found that appropriate embryonic eggs could provide hepatocytes with a small amount of non-parenchymal liver cells. It is well known that heterotypic cell interactions are required for the phenotypic stability of the parenchymal cells as well as for proper liver function. Several studies have highlighted the importance of hepatic function of hepatocytes when supported with non-parenchymal liver cells [40]. Additionally, multi-step digestion and washing could limit non-parenchymal cells to a low level so that there was no need to eliminate them by other treatments. The immunofluorescent assay in our study measured the secreted ALB of isolated hepatocytes as a marker for protein synthesis function of cultured hepatocytes as well as examined the purity of isolated hepatocytes. Furthermore, detection of *alb* and *afp* at mRNA level was another method to confirm the hepatocytes. The ability of hepatic glycogen storage and the activity of G6PDH were both tested to ensure hepatocytes retained their biological function. Given the above, a method for primary culture of duck embryo hepatocytes with high viability, excellent purity and maintained function was established, that was characterized by quickness, ease of operation and low cost and easily available material. And this primary culture technology can also provide the basis for other primary cells culture.

Choline is an essential nutrient for animal and human and the primary fates of choline are generation of phospholipids as main component of cell membranes through phosphorylation and production of S-adenosyl-L-methionine as a donor of methyl groups by oxidation action [41,42]. In addition, there is evidence suggesting that low choline diets develop fatty liver and liver damage [43,44]. Exogenous choline is also needed by many species of animal cells for normal growth *in vitro*. Based on the method of isolation and culture of primary hepatocytes, we explored lipid metabolic alterations in choline-deficient duck primary hepatocytes model. As expected, choline deficient group occurred accumulation of TG and cholesterol in hepatocytes. The reasons might be the decreased PCs, which is necessary for the packaging and export of TG, resulting in reduced secretion of very low-density lipoprotein due to the absence of choline [45]. Our data showed that the concentrations of TG and T-CHO in hepatocytes were significantly increased in choline-deficient model after the treatment for 12 h. At 24 h, the content of TG in choline-deficient treatment significantly remained higher than that in control group and the content of T-CHO in choline-deficient treatment was lower but not significant. The results were consistent with early studies and may provide NAFLD model for further research, which also confirmed that duck primary hepatocytes were isolated and our optimization method for it was constructed successfully.

Previous analysis of possible molecular mechanisms for choline metabolism has largely focused on gene expression rather than gene structure. It is reported that DNA mutation is not the only cause of cancer, and AS can also be the killer [46]. New or specific isoforms and disorder isoforms derived from AS are closely related to tumorigenesis and cancer progression [47–49]. Accordingly, it is suspected that whether AS events altered in the choline deficient group. Nowadays analysis of AS is facilitated by RNA-seq technology. The results showed that AS has higher frequency in CD treatment group. AS is the process by which splice sites in precursor messenger RNA (pre-mRNA) are differentially selected to produce multiple mRNA and protein isoform. KEGG pathways in CD group indicates that AS related genes were significantly enriched in genetic information processing including folding, sorting and degradation pathway, replication and repair pathway and translation pathway, which may be the primary regulator in post-transcriptional level. Additionally studies reported that AS had differential distribution patterns in various species [50]. In plants, RI is the predominant case of AS, while not in animals. In the line with studies on human, SE is the major case of AS in our study, which occupies more than 70% of total AS events. One reason for that is spliceosomal components differ in different species. Furthermore, different patterns of AS has varied effects on individuals’ function. Indeed, SE are more prone to creating and changing the protein function due to the deletion of functional motif [51,52]. Moreover, accumulating evidence has suggested that mutations and/or altered expression in splicing regulators and aberrant splicing alterations in the obesity-associated genes are often linked to humans’ diet-induced obesity and metabolic dysregulation phenotypes [53]. It is well known that choline was involved in regulating the host’s lipid metabolism. We focus on lipid metabolism related genes involved in significant AS, which represents 16 genes enriched in glycerophospholipid metabolism. The findings echo the results of that lysophosphatidylcholine, PC and choline expression were closely related to glycerol phospholipid metabolism [54]. Of note, two of these 16 genes called *pla2g3* and *pla2g4a*, belong to the family of phospholipase A2 proteins, with the different AS including A3SS, and SE. *Pla2g3* encoding secreted PLA2 protein, while *pla2g4a* encoding cytosolic PLA2 protein, indicates that various AS events in the regulation and enrichment of the metabolism processes. The analysis of rMATS of *pla2g3* showed A3SS existed with ΔPSI = 1 in CD group. AS could undergo modulation and close interaction with genetic and epigenetic machinery. Choline as a kind of methyl donor, participates in the methylation-dependent biosynthesis of DNA and RNA, can alter methylation status of *pparα* gene reflecting on hepatic lipid metabolism [45] and the site of methylation of gene is closely associated with AS [55]. We speculate that predominant splicing sites may be lost due to aberrant methylation level of genes. Our finding provided a novel insight into various AS in the metabolism role of choline.

## CONCLUSION

Overall, we performed an optimized enzymatic digestion technique for primary hepatocytes from duck embryos, obtaining duck primary hepatocytes with high purity and biological activity checked both morphologically and functionally, which also provide the basis for other primary cells culture. Then, NAFLD model was constructed in choline-free medium and AS altered significantly in choline-deficient model, prompting a novel insight into relevance between nutrients and gene structure.

## SUPPLEMENTARY MATERIAL

**Supplementary Figure S1**. Hepatocytes were isolated from different ages Pekin duck. **Supplementary Figure S2**. Hepatocytes viability under different choline levels treatments. **Supplementary Table S1**. Five basic types of alternative splicing (AS) events occurred in each sample **Supplementary Table S2**. AS events involved related lipid metabolism in choline-deficient differed from that in control for details
