# Supplementary material for: Severe choline deficiency induces alternative splicing aberrance in optimized duck primary hepatocyte cultures
Source: Anim Biosci. 2022 May 2;35(11):1787–99. doi: 10.5713/ab.22.0051 (PMC9659469; doi:10.5713/ab.22.0051)
Supplement: Supplementary file 1 [file ab-22-0051-suppl.pdf]

Supplymentary 1

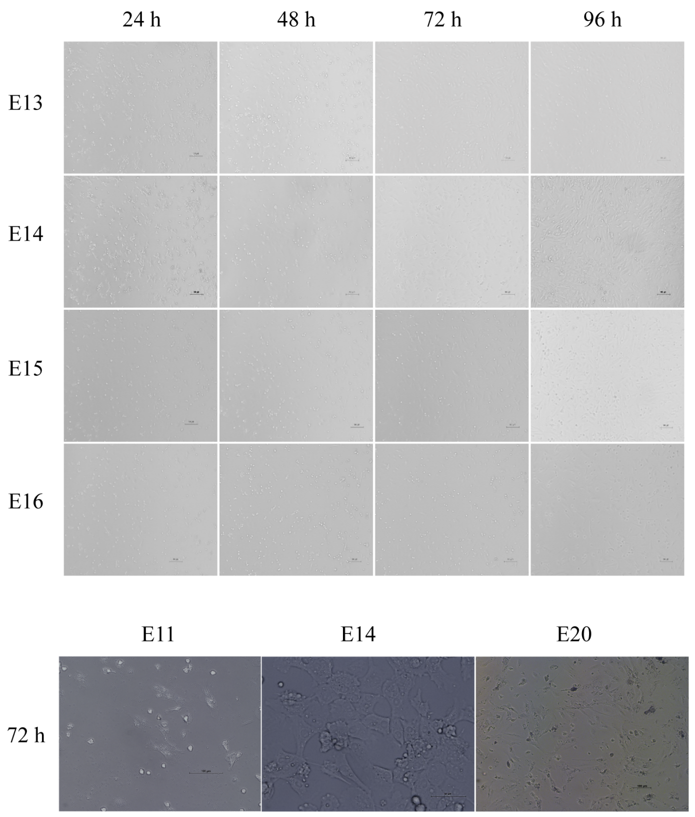

Figure.S1 Hepatocytes were isolated from different ages of Pekin duck.

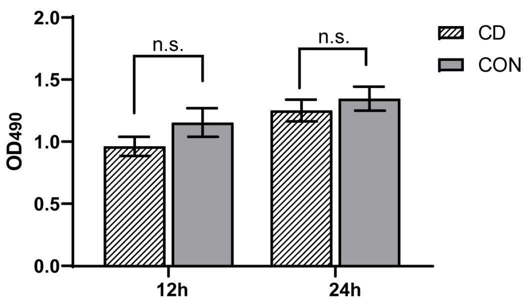

Figure.S2 Hepatocytes viability under different choline levels treatments

Supplementary 2 (S2)

Table S1 Five basic types of alternative splicing (AS) events occurred in each sample.

AS Types	CON Group (events)			CD Group (events)			*P* value
SE	22201	22232	22251	26801	26850	26830	0.000
RI	752	773	772	815	821	833	0.003
A5SS	1567	1561	1576	1656	1657	1651	0.000
A3SS	2405	2397	2417	2518	2530	2555	0.000
MXE	3972	3970	3999	5017	5053	5052	0.000
Total	30897	30933	31015	36807	36911	36921	0.000

CD means the samples in choline-deficient model and three parallel samples conducted in each treatment, as CON means control group.

Table S2 AS events involved related lipid metabolism in choline-deficient differed from that in control for details.

AS ID	Gene ID	Gene name	Gene description	Novel AS	Chr	Strand	Diff significant	IncLevelDiff(NORMAL/CD,ΔPSI)	P Value Junction Count Only	FDR JunctionCountOnly
SE_16726	gene7258	HMGCLL1	3-hydroxymethyl-3-methylglutaryl-CoA lyase like 1, transcript variant X1	yes	3	+	yes	0.384	0.000418	0.030107
SE_16724	gene7258	HMGCLL1	3-hydroxymethyl-3-methylglutaryl-CoA lyase like 1, transcript variant X1	no	3	+	yes	-0.468	0.000484	0.033373
SE_20683	gene12749	TECR	trans-2,3-enoyl-CoA reductase, transcript variant X1	yes	8	-	yes	-0.339	5.47E-08	5.43E-05
SE_7996	gene584	DGKH	diacylglycerol kinase eta	yes	1	-	yes	-0.529	2.6E-07	0.000186
SE_25644	gene1141	AGPAT3	1-acylglycerol-3-phosphate O-acyltransferase 3, transcript variant X2	no	1	+	yes	0.262	0.000234	0.019832

AS ID	Gene ID	Gene name	Gene description	Novel AS	Chr	Strand	Diff significant	IncLevelDiff(NORMAL/CD,ΔPSI)	P Value Junction Count Only	FDR JunctionCountOnly
SE_17302	gene12420	PLA2G4A	phospholipase A2 group IVA, transcript variant X3	no	8	+	yes	-0.091	5.07E-08	5.26E-05
SE_17303	gene12420	PLA2G4A	phospholipase A2 group IVA, transcript variant X3	no	8	+	yes	-0.615	2.87E-11	1.64E-07
SE_17300	gene12420	PLA2G4A	phospholipase A2 group IVA, transcript variant X3	no	8	+	yes	-0.194	0.000139	0.013482
MXE_262	gene11889	LOC101802865	sterol 26-hydroxylase, mitochondrial	no	7	+	yes	0.294	0.001509	0.034249
MXE_4810	gene5316	PTDSS1	phosphatidylserine synthase 1, transcript variant X1	yes	2	+	yes	0.119	0.000446	0.012999

AS ID	Gene ID	Gene name	Gene description	Novel AS	Chr	Strand	Diff significant	IncLevelDiff(NORMAL/CD,ΔPSI)	P Value Junction Count Only	FDR JunctionCountOnly
MXE_2813	gene9770	TKFC	triokinase and FMN cyclase, transcript variant X4	no	5	+	yes	0.245	2.21E-05	0.001246
A3SS_708	gene19086	ACOT8	acyl-CoA thioesterase 8	no	21	-	yes	0.127	0.001457	0.041449
A3SS_3725	gene17017	PLA2G3	phospholipase A2 group III, transcript variant X1	no	16	+	yes	1	9.29E-13	2.35E-09
A3SS_1133	gene9070	GPD2	glycerol-3-phosphate dehydrogenase 2, transcript variant X1	no	5	+	yes	-0.667	0.000122	0.006435
A3SS_752	gene21060	LIPG	lipase G, endothelial type	no	Z	-	yes	-0.065	0.001629	0.044838

AS ID	Gene ID	Gene name	Gene description	Novel AS	Chr	Strand	Diff significant	IncLevelDiff(NORMAL/CD,ΔPSI)	P Value Junction Count Only	FDR JunctionCountOnly
A5SS_2007	gene7726	ELOVL6	ELOVL fatty acid elongase 6	no	4	+	yes	0.176	6.4E-06	0.000705
A5SS_298	gene12100	LOC101792912	cytochrome P450 2J2-like, transcript variant X3	no	8	+	yes	-0.707	8.89E-07	0.00021
A5SS_2100	gene16277	PEMT	phosphatidylethanolamine N-methyltransferase, transcript variant X2	no	15	+	yes	0.1	2.89E-05	0.001836
A5SS_1080	gene11740	GLB1L	galactosidase beta 1 like, transcript variant X1	no	7	+	yes	0.076	0.000222	0.008534
